# Trading quality for relevance: non-health decision-makers’ use of evidence on the social determinants of health

**DOI:** 10.1136/bmjopen-2014-007053

**Published:** 2015-04-02

**Authors:** Elizabeth McGill, Matt Egan, Mark Petticrew, Lesley Mountford, Sarah Milton, Margaret Whitehead, Karen Lock

**Affiliations:** 1 Department of Health Services Research and Policy, London School of Hygiene & Tropical Medicine, NIHR School for Public Health Research, London, UK; 2 Department of Social and Environmental Health Research, London School of Hygiene & Tropical Medicine, NIHR School for Public Health Research, London, UK; 3 Stoke-on-Trent City Council, Stoke-on-Trent, UK; 4 Department of Public Health and Policy, University of Liverpool, NIHR School for Public Health Research, Liverpool, UK

**Keywords:** QUALITATIVE RESEARCH, Public health, policy

## Abstract

**Objectives:**

Local government services and policies affect health determinants across many sectors such as planning, transportation, housing and leisure. Researchers and policymakers have argued that decisions affecting wider determinants of health, well-being and inequalities should be informed by evidence. This study explores how information and evidence are defined, assessed and utilised by local professionals situated beyond the health sector, but whose decisions potentially affect health: in this case, practitioners working in design, planning and maintenance of the built environment.

**Design:**

A qualitative study using three focus groups. A thematic analysis was undertaken.

**Setting:**

The focus groups were held in UK localities and involved local practitioners working in two UK regions, as well as in Brazil, USA and Canada.

**Participants:**

UK and international practitioners working in the design and management of the built environment at a local government level.

**Results:**

Participants described a range of data and information that constitutes evidence, of which academic research is only one part. Built environment decision-makers value empirical evidence, but also emphasise the legitimacy and relevance of less empirical ways of thinking through narratives that associate their work to art and philosophy. Participants prioritised evidence on the acceptability, deliverability and sustainability of interventions over evidence of longer term outcomes (including many health outcomes). Participants generally privileged local information, including personal experiences and local data, but were less willing to accept evidence from contexts perceived to be different from their own.

**Conclusions:**

Local-level built environment practitioners utilise evidence to make decisions, but their view of ‘best evidence’ appears to prioritise local relevance over academic rigour. Academics can facilitate evidence-informed local decisions affecting social determinants of health by working with relevant practitioners to improve the quality of local data and evaluations, and by advancing approaches to improve the external validity of academic research.

**Strengths and limitations of this study**

- This study advances research into evidence-informed policymaking with a novel focus on local-level decision-making in policy areas outside the health sector that affect the social determinants of health and health inequalities.
- Participants were recruited from international settings and two distinct UK regions.
- This paper provides practical recommendations on how academics can more fruitfully engage with and support local-level decision-making.
- The sample size was relatively small and participants were purposively sampled within one particular policy area (the built environment).
- Participants may have agreed to take part in the study because of an a priori interest in evidence utilisation in decision-making.

## Introduction

Social, economic and environmental factors contribute to population health and health inequalities, suggesting a need for public health strategies that extend their scope beyond health service delivery.^1–3^ Following this rationale, the WHO's Ottawa Charter, 1986, called for action on health ‘in all sectors and at all levels’ of policy.^4^ Local government services and policies affect health determinants across many sectors such as planning, transportation, housing and leisure.^5^ As such, the local policy level is crucial for multisectorial strategies aiming to deliver *healthy public policy*.^6^ ^7^

Alongside calls to make public health decision-making multisectorial, there have been concurrent calls for ‘evidence-based’ or ‘evidence-informed’ decisions.^8^ ^9^ Both rhetoric and specific initiatives associated with ‘evidence informed policy and practice’ have been taken up globally, encouraged by organisations such as WHO and the World Bank.^10^ ^11^ This response is prominent within the public health community,^12^ ^13^ perhaps reflecting strong pedagogical and institutional links with medical disciplines that have advanced the view that treatments should be evidence-based. Outside the public health profession, the salience and utilisation of an ‘evidence-informed’ approach may vary across professions.^14^ ^15^ Different sectors have their own associated research traditions and sources of knowledge, leading to different conceptualisations of what evidence is and how it can be applied.^16^

National and international initiatives have encouraged the production and synthesis of research evidence to inform decision-making across sectors,^17–19^ and to better understand the processes by which evidence contributes (or fails to contribute) to policy and practice.^20–25^ A systematic review found that barriers to and facilitators of evidence-informed policy have been the subject of research in approximately 60 different countries worldwide, including many low-income and middle-income nations.^26^ Evidence use outside the health sector has also been studied in Europe, North America and Australasia.^14^

However, a counter-current in the literature and policy debates has challenged the concept of evidence-informed policy. Some commentators have questioned the usefulness and appropriateness of evidence-informed policy in general.^27^ Types of evidence utilisation have been described that include strategic citing of academic work (or the lack of it) to provide posthoc justifications for policies, decisions and ideologies. Such descriptions suggest a scepticism that research findings are incorporated into decision-making through simple, linear processes of ‘knowledge translation’.^9^ ^28^ This has led some commentators to emphasise the need to better understand the ‘nexus’ where policy, practice and research meet by identifying points of contact, barriers and strategies to encourage joint working and cooperation.^29^ ^30^ Much of this research activity relates to national and regional-level decision-making. We know less about evidence utilisation in local government across a range of sectors.^31^

Recent developments affecting local-level public health administration in England illustrate the need to know what different sectors in local government understand by evidence informed decision-making.^32^ In April 2013, after 40 years in the National Health Service (NHS), the statutory public health services returned to local government, suggesting that public health professionals share (in theory) greater organisational links with decision-makers delivering on local services across other sectors.^33^ While criticised by some,^34^ this transfer has been advocated as an opportunity to encourage multisectorial working and more fully incorporate the social determinants of health approach into public health policy and practice.^35^ ^36^

As the public health agenda continues to be integrated into local government, we argue that researchers need to know more about the types of evidence and information relevant to and sought by local government decision-makers.^14^ ^31^ ^37^ In this paper we focus on local practitioners working on the built urban environment. We define the built environment broadly to ‘comprise urban design, land use, and the transportation system, and encompasses patterns of human activity within the physical environment’ (p.65).^38^ The emphasis on the built environment underscores a broad conceptualisation of the social determinants of health and recognises the impacts that urban design, transportation, housing systems and land use may have on population health.^39–42^ This qualitative study was undertaken to better understand how decision-making occurs in these areas of local government. Specifically, it focuses on how information and evidence are defined, assessed and utilised by local decision-makers working in areas related to the design, planning and maintenance of the built environment.

## Methods

This qualitative study is based on three focus groups held in 2013. Local government professionals were asked to participate in focus groups that aimed ‘to discuss and to learn more about how policymakers in local, city or regional government use information and evidence in their policy-making processes in built environment sectors’. Participants were purposively recruited through professional contacts and snowballing. For pragmatic reasons influenced by the researchers’ locations, two groups represented two UK regions (London and North West England), while the third was an international group (city representatives from Brazil, USA, Canada, England). We planned that the groups could capture perspectives across and beyond the UK.

The focus group facilitator (MP) used a topic guide with open questions and prompts about how participants conceptualise and use evidence in their work. Our approach replicated previous studies exploring national policymakers’ and researchers’ perspectives on evidence use.^43^ ^44^ All discussions were audio recorded and transcribed.

A thematic analysis method^45^ ^46^ involved developing initial coding structures agreed by two authors following full readings of the text, and drawing on existing work on evidence and policy.^8^ ^9^ Inductive coding was then undertaken by EM in consultation with the other coauthors as additional themes were identified through reading transcripts. Emerging findings were discussed by the research team and themes were applied to the data and refined throughout the analysis. Coding was double-checked by a second researcher. NVivo10 was utilised to aid in data management and coding.

Three overarching questions framed the analysis: (1) Which types of evidence did participants consider useful for their practice? (2) Whether their status as built environment practitioners influenced their approach to evidence? (3) Whether their status as local practitioners influenced their approach to evidence? As the analysis progressed, we selected emergent themes which we report in the findings section and then return to the three overarching questions in the discussion.

## Results

A total of 15 senior local government decision-makers participated in the focus groups; their respective work roles are presented in table 1.

**Table 1:** Participants’ work roles

	International	London	North West, England
Planning manager	1	2	3
Urban designer	2	1	0
Elected official	2	0	0
Director or commissioner of built environment services	1	1	0
Housing manager	0	0	1
Leisure services manager	0	0	1

The emergent themes we identified are described below.

### Multiple concepts of evidence

Participants across the three workshops juxtaposed a narrow view of health research as ‘pure science’ (ie, driven by high quality empirical research) with the built environment as a form of creative ‘art’, a philosophy or in one participant's words, a ‘theology’. Participants sometimes compared themselves against the archetype of the *scientist*, variously referred to as ‘medical’, ‘biomedical’ or ‘pure science’. An international participant illustrated this point, implying that the need to make evidence-informed decisions was traditionally perceived to be a barrier to creativity, but the participant also provided the opinion that architecture and related professions were gradually moving towards more evidence-informed approaches:

I think if you're talking about the design professions, architects, in particular, have never felt constrained by the need for evidence to do things…we don't care. So I think it's come slowly to the design professions, but it's growing. [Commissioner, International]

Group discussions did tend to suggest that practitioner roles could combine artistic and scientific elements. However, discussions also referred to the visual arts, emphasising the physicality and visibility of urban design and planning. Evidence could still be relevant to practice, but it could be image rather than text based. Architectural drawings, simple sketches, photographs and design plans all become valuable and instructive sources of information. Qualitative research could also be highly valued as a means of assessing the opinions and experiences of different stakeholders.

when you get down to the built environment, it's a, it's not a pure science, right? It's science and it's art, and so therefore quantitative information is one aspect of it. But if you look at a great, healthy city…there's a qualitative aspect of it in there. [Urban Designer, International]

We found participants from all three focus groups gave examples of quantitative and qualitative data used to inform their decisions, but again the kind of evidence they referred to was not simply analogous to academic research. This ‘evidence’ could include routinely collected data, specially commissioned surveys, local maps and associated geographic information systems (GIS) data, evidence-informed guidelines, anecdotes, case studies, mathematical models and academic research. As a planning manager described:

…it could be anything from having the sort of latest population census, the latest population forecast, to knowing, um, or having information around, say, assessing how much housing land you need…it could be, say, around having studies that have been done around the benefits from, say, putting green infrastructure, which could be a whole range of things, everything from health to, to preventing flooding to recreation to, to whatever. Um, so it can be, you know, from the sort of statistical side. Through forecasting, through, um, best practice studies. Um, it can be a whole range of things from our perspective. [Planner, London]

### ‘Viability’ versus outcomes

Participants frequently spoke about the need to demonstrate the ‘viability’ of interventions and services, with the focus on whether initiatives could be delivered, sustained and accepted by stakeholders and users. The demand for evidence on ‘viability’ was sometimes said to come from national government.

Increasingly the government is putting more evidence on, onus on, that viability side of things. What you can actually deliver. Rather than what you actually need. [Planning Manager, North West, England]

Interventions were considered non-viable if they were believed to be incommensurable with national legislation, finance, contractual obligations and government endorsed guidance for best practice or if they were unacceptable to local stakeholders, such as delivery partners, politicians and the public. Data that might provide evidence of acceptability could come from market research, surveys and routine data on service participation rates. Pilot programmes were viewed as particularly valuable because they gave stakeholders an early opportunity to see how an intervention could be delivered without making large-scale commitments.

And what's actually really powerful about [pilot projects] is that, one, they're small scale, and two, by doing it as a pilot project, what they're able to do is not, basically not freak everyone out, right? [Urban Designer, International]

Cost was also identified by participants in each focus group as crucial to demonstrating that an intervention could be sustained. A strong emerging theme focused on the perception that the current economic climate led to budget constraints and straitened financial circumstances, which increased political pressure to demonstrate value for money.

I'm being asked more questions around monetisation than we were in the past. And I think particularly with the link to viability of development. [Urban Designer, London]

Participants said they used indicators, such as output delivery, economic development indices and growth surveys, to make the case for economic benefits that may have resulted from their work—but there was no mention of using or commissioning more formal cost-benefit studies. The following comment by a practitioner from the North West illustrates how participants framed their discussions of economic benefits in a narrative that associated successful delivery of project outputs with plausible (but not necessarily measured) impacts on the local economy:

He [an elected official] will be interested in the job creation, now, and the number of homes, number of completions, so there's a lot of performance tracking, there's economic development activity. And that feeds into viability, you know. [Planning Manager, North West]

In contrast to the repeated reference to ‘viability’, participants from the three focus groups tended not to emphasise evidence of longer-term intervention outcomes. Reasons given for this included the challenge of measuring and attributing long-term intervention effects, difficulties in accessing and interpreting (sometimes contradictory) research evidence, as well as a perceived lack of political interest in evidence of such outcomes.

I think the outcomes is the difficult one. It's how do we attribute the outcomes to our housing intervention. [Housing Manager, North West England]

Because both public health and built environment are such long term investments, right, in the life of a community and so to build the evidence…is tricky. [Urban Designer, International]

Actually there's a load of really good health outcomes in terms of looking at housing intervention, you know…But it's a non interventionist government who actually don't really believe in evidence policy making, really, to be honest with you. [Planning Manager, North West]

However, when discussing their own practice, some participants did identify occasions where there was an opportunity to consider academic evidence of intervention outcomes, particularly during consultations, assessments and drawing up of guidelines and standards. For example, one participant described an occasion ‘when we were working on the active design guidelines we found academics who were actually doing evidence-based research’ [Councillor, International], outcomes from which were then incorporated into the guidelines.

### Locally relevant evidence

Participants placed knowledge about their local area at a premium. Quantitative data aggregated at the level of the local authority helped practitioners compare their authority against its’ neighbours or against national indicators. Microlevel data, for example, street or address level which could be quantitative, qualitative or anecdotal, gave practitioners information about specific buildings, intervention/service delivery points or specific localities associated with certain problems or issues of interest. For example, one participant was involved in a local initiative that:

…essentially took GIS mappings of the city and looked at where the highest rates of obesity and diabetes were, and then we looked at…where supermarkets were located. [Urban Designer, International]

This fine grain local data was often not academic in origin, although academics may have been involved in its production. For instance, a participant from a London local authority described the UK Government’s Decennial Census as the initial starting point for much of their area level statistics. Local authorities also conducted their own data collection:

We'd do a housing needs survey, and that should show us how much affordable housing we're short of. So then we would write a policy to say we want so many percentage of each development to be affordable. [Planner, North West]

Besides using formally-collected quantitative data, practitioners drew on personal local knowledge. As local practitioners work within relatively small geographical boundaries, they are able to build up detailed experience-based understanding (based on a kind of participant observation) of their own area's geography, organisations, processes and issues.

In addition, practitioners from all three groups also saw the value of ‘case studies’ from areas similar to their own.

…case studies are really valuable in terms of being able to show how a policy can be put in place and, and you can get an outcome that you're actually looking for. [Planning Manager, London]

Case studies were described in different ways: they could be as simple as an anecdote or a site visit, or they could involve more formal attempts to collect data about an intervention's delivery, acceptability, cost and (sometimes) outcomes. The appeal of both formal and informal case studies lay in their perceived value in convincing local practitioners that a particular intervention had been successfully delivered in a similar area, by professionals similar to themselves and working within a similar regulatory and financial framework. This provided some assurance that the intervention could be delivered successfully in their own area.

[It helps] to be able to say, well, this city of a similar size in a similar country, and preferably a neighbouring borough did it [i.e. delivered the intervention]. And not only did it achieve some of the things we thought it would, in terms of health outcomes or employability, whatever, but also, it didn't result in massive political damage. [Elected Official, International]

In contrast, participants were less willing to accept evidence relating to interventions delivered in contexts perceived to be different from their own, including those reported in published academic studies in international journals.

But I think often, I mean, we're talking about, you're talking about overseas evidence, and often the problem, I think, is where people use overseas evidence is that the planning systems are so different it's not easy to track for a local authority or another organisation to see how you can actually maybe translate that back into the British situation. [Planning Manager, London]

Hence, academic research could be discounted because of perceived problems with its external validity (specifically, its relevance to the participants’ own local area and/or practice), irrespective of the internal validity of the evidence. The local relevance of evidence was generally seen to be more importance than its methodological rigour.

## Discussion

We have used focus groups to explore how information and evidence are defined and utilised by local decision-makers working in design, planning and maintenance of the built environment. In particular, we aimed to explore (1) the types of evidence that non-health sector local practitioners consider useful for their practice; (2) whether practitioners specialising in the built environment have a particular view of evidence influenced by their professional sector and its culture; and (3) whether practitioners working at a local level may have a particular view of evidence that is related to the spatial scale at which they operate.

### Types of evidence

Practitioners refer to various types of evidence besides that found in academic publications. This evidence includes ‘routine data’ produced by public and private sector organisations, sometimes with academic involvement, such as surveys, maps and audit data. Less ‘academic’ sources of knowledge include practitioners’ first hand experiences and anecdotal evidence. Practitioners also find case studies, defined in different ways, particularly useful for demonstrating that an intervention can be successfully delivered in a similar context.

Previous research on models of evidence utilisation (often at a national level) has suggested that decision-makers may use academic research strategically to provide posthoc justifications for policies, actions and inaction.^9^ ^28^ Our findings do not contradict these earlier works, but they do suggest the possibility that a more broadly conceived view of ‘evidence’, as described above, can help reveal how local practitioners do routinely use evidence to inform decisions—but the evidence is not necessarily findings from international peer review journals.

### Built environment practitioners and evidence

Built environment practitioners present narratives intended to explain why academic evidence is not always incorporated into decisions. One kind of narrative juxtaposed participants’ preference for a broad approach to knowledge (including philosophy, art and design) to a narrowly conceived depiction of ‘pure’ empirical science. Participants’ self-presentations also tended to emphasise artistic and creative aspects of their work, which they again contrasted with empirical science.

Sociological literature on science, health and the health profession has problematised definitions and distinctions relating to science and other epistemologies.^47–49^ Our findings highlight built environment practitioners’ self-identification with the visual and creative arts and humanities, and suggest that this can sometimes make empirical research evidence seem less relevant to them.

### Local practice

Local intelligence is crucially important to local practice—an obvious statement, but one that has far-reaching implications for evidence-informed decision-making. Even though academics often take seriously the need to provide evidence that can inform policy and practice, localism can be a barrier to knowledge translation. Local practitioners question whether articles published in international academic journals are necessarily relevant to their own context. They may consider locally specific routine data, locally commissioned surveys and qualitative data collection to be more useful because these data have a direct and obvious relevance to the specific settings within which local practitioners work.

### Research implications

While the authors of this paper support the need for evidence-informed decisions, we would add that in order to deliver on evidence-informed policy and practice, more academics need to look at the evidence needs of local practitioners and tailor research to better meet those needs. We are not the first to advance this point of view. Previous discussions have often been framed around the kinds of evaluation questions practitioners find most useful (eg, ‘what works, for whom and in what context?’),^50^ the need for intervention evaluations to identify the theories and/or mechanisms of change,^51^ and around debates for and against experimental and quasi-experimental research methods when evaluating interventions in complex settings.^52^ ^53^ In contrast, we have focused on exploring what local practitioners outside the health sector themselves consider to be useful evidence relevant to their everyday work.

The findings suggest why local practitioners working beyond the health sector may not always choose to prioritise academic research outputs among the different types of knowledge relevant to their work. However, they also highlight ways in which academics may help inform local decision-making through involvement in developing and analysing locally relevant data, improving practitioners’ skills for evaluation, and paying more attention to contextual issues, external validity and the transferability of study findings.

### Practice implications

Practitioners’ prioritisation of local knowledge, including quantitative and qualitative data, and experiential knowledge of a locality seems to be rational and unavoidable. Local government decision-makers face a complex set of limitations, levers and discretionary powers that frame what they may and may not be able to achieve within the political, legislative and financial landscape within which they operate. Given this complexity, practitioners may feel sceptical about how academic studies from a wide range of (often poorly described)^54^ contexts and settings can help them with their particular jobs.

However, practitioners do agree that case studies can be particularly useful, especially when they describe success stories delivered in similar contexts to their own practice. Case studies have also attracted the interest of some academics interested in evaluating social interventions.^55^ ^56^ We, therefore, see an opportunity for more practitioners and academics to work together to produce methodologically robust case studies of local innovation. These could take on the character of informal pilot studies—providing practitioners working in similar contexts with an opportunity to assess whether an intervention might be both viable *and effective* within their own area. Academics, who increasingly need to demonstrate that their research has been translated into practice and has ‘impact’, stand to benefit from this symbiotic relationship.

### Limitations

Focus group research presents the researcher with examples of how practitioners interact and construct narratives with their peers, and this may differ to how they would present their opinions if interviewed individually or indeed any opinions they might not want to voice. In this particular study we noticed a general consensus within and between groups. This may reflect genuine consensus, but it could also reflect possible methodological limitations: for example, self-selection (ie, participants agreeing to take part because they were interested in evidence-informed policy); researcher bias (participants framing responses around their beliefs about what the researchers wanted to hear); and group dynamics among professionals who may tend to seek out consensus. The groups were small and purposively sampled. We focused on built environment practitioners because of the well-established theoretical and empirical associations between built environment and public health, but we assume that this selection influenced our findings.

## Conclusion

For some time, commentators with an interest in public health have advanced the view that decisions should be informed by the best available evidence. It is sometimes assumed that ‘best available evidence’ is synonymous with methodologically robust academic research. However, nearly two decades ago, Nutbeam pointed out that evidence-free policy was in part caused by ‘policy-free’ evidence: academic research that fails to adequately understand and address the needs of decision-makers.^57^ This study suggests a similar relationship between evidence and local practice outside the health sector relevant to the social determinants of health. From this study we suggest that built environment practitioners in local authorities do try to base their decisions on the best available evidence—but their conceptualisation of ‘best’ prioritises relevance over rigour and external over internal validity. In particular, they value knowledge about their local area, be it quantitative, qualitative or experiential. They also value stories of an intervention or service being successfully delivered in a similar setting by practitioners working with the same constraints as themselves. Academics can (and some do) address the problem of ‘policy or practice-free research’ by working with local practitioners from within and more crucially, beyond the health sector to improve the quality of evidence sources that are most valuable to local decision-making influencing the social determinants of health.^58^ Bringing greater rigour to local data analysis and to case studies of specific local initiatives provide a means, we suggest, of optimising rather than choosing between the internal and external validity of evidence.

**Key messages**

- Built environment practitioners in local authorities apply the word ‘evidence’ to a variety of knowledge sources including case studies.
- Practitioners seek evidence of viability, a conflation of terms relating to the feasibility of intervention delivery and sustainability.
- Emphasis is placed on immediate outputs and intermediate outcomes; evidence of long-term outcomes, including health outcomes, may be seen as unattainable.
- Local knowledge is vital to local practice. Academic evidence is frequently irrelevant to practitioners' local contexts.
- Academics could support work on the social determinants of health in local authorities more effectively by co-producing research with local practitioners, by developing geographical data at local authority level, and by improving local evaluation and research capacity through training.
